# Generalized environmental fear hypothesis and the effects of schematic restructuring in autism

**DOI:** 10.3389/fpsyt.2026.1725265

**Published:** 2026-02-24

**Authors:** Alan Long, Nishant Revanur, Kyra Crowder

**Affiliations:** 1 Department of Psychology, Arizona State University, Tempe, AZ, United States; 2 Department of Public Health, Aggie Research Programs, Texas A&M University, College Station, TX, United States

**Keywords:** ASD, autism, cognitive psychology, schematic restructuring, relational mapping

## Abstract

Current Models of Autism Spectrum Disorder (ASD) are very complex and exploratory in nature, it is the general consensus that there is not one underlying cause of autism. This article seeks to contest that claim by supporting a hypothesis that accounts for multiple, if not all, subgroups with a single common factor. At the same time, this hypothesis would lead to the use of Cognitive Behavioral Therapy (CBT) for researching therapeutic methods for people with ASD, and this article outlines why that is not advisable. It is hypothesized that upon realization of awareness in the womb, people with ASD conditioned a fear response to their environment. This fear response generalized upon the realization of new cognitive awareness, leading to the symptoms of ASD. In regards to CBT, it is hypothesized that schematic restructuring (a result of CBT) can lead to symptoms associated with CPTSD and schizophrenia. We further hypothesize that addressing schema in reverse order of acquisition will reduce the risk hypothesized to be associated with CBT. Drawing on schema theory, developmental stages, and neurobiology this article argues for the validity of these hypotheses. The implications of these models are vast, not only for the field of Autism research, but for the field as a whole. While some of the implications may seem negative for the field (CBT), the authors ultimately support the use of CBT for many situations other than ASD.

## 1 Introduction

Autism Spectrum Disorder (ASD) is diagnosed in 1 in 32 people aged 8 years (1). While many people with ASD value their neurodivergence, there are also many suffering from their symptoms. Furthermore, Cognitive Behavioral Therapy (CBT) is frequently used to treat a variety of disorders as well as employed to assist people in overcoming “maladaptive” schema (2). While this hypothesis does not draw significantly from any one model, many were used to refine our understanding of the symptoms of ASD (3–8). It is through their effort that this article is able to hypothesize on a commonality between the symptoms of ASD.

The Generalized Environmental Fear Hypothesis is as follows: It is hypothesized that upon realization of awareness in the womb, people with ASD conditioned a fear response to their environment. This fear response generalized upon the realization of new cognitive awareness, leading to the symptoms of ASD. While our hypotheses regarding CBT are as follows: it is hypothesized that schematic restructuring (a result of CBT) can lead to symptoms associated with CPTSD and schizophrenia. We further hypothesize that addressing schema in reverse order of acquisition will reduce the risk hypothesized to be associated with CBT. Finally, it is hypothesized that due to the age of the “fear schema” in people with ASD, you can expect significant schematic restructuring should it be addressed before newer schema.

## 2 Introduction to generalized environmental fear hypothesis for autism

In the functioning of schema, there must be some form of relational mapping. To support this claim, this article will work backwards from Deanna Kuhn’s Levels of Epistemological Understanding starting at stage three and add a zero stage to her theory (9). In this stage children become aware of opinions; the relationships here are “This person thinks opinion about my reality.” Stage two, the child becomes aware of facts. “This statement applies/doesn’t apply to my reality.” Stage one, the toddler becomes aware of reality. “This is my reality.” Stage zero, the infant has a conception of reality. “My reality.” In different terms, “At this location this is sensed.” Stage zero is representative of sensation to externalities during which infants define “my reality.” The order of acquisition indicates that at each level we are adding a relational map as opposed to a new cognitive skill.

Studies have shown that emotion, location, and state of mind have positive correlations with the recall of formed memories (10–13). Due to these formations, we can assume that schemas contain relational data in the form of rational and emotional positioning between items and that the relational data is utilized in memory recall and formation. This is confirmed by generalization between schemata. While in the presence of aversive stimulus A, stimulus B is presented. Because of the emotional state, B is recorded with “fearful” relational data. As stimulus C is presented with similarities to stimulus B, a schema forms connecting stimulus C to stimulus B and relational data from B to A is recalled (14). Due to this, fears are relational data for schemas.

This is the foundation of Generalized Environmental Fear Theory for Autism. Upon the realization of an environment, there is a potential for a fetus to develop an environmental fear. Due to the recognition that fears are relational data on schema and the studies showing that overgeneralization was more likely to occur the younger the child is, we can expect this environmental fear to generalize upon the arrival of new cognitive experiences (15).

Returning to Deanna Kuhn’s Levels of Epistemological Understanding (9), the environmental fear is representative of our theoretical stage zero. Upon the realization of “their reality” in the first stage, the fear will generalize to their reality, leading to reduced sensorimotor development (16). This is also shown by reduced eye fixation in 2–6 month old infants later diagnosed with ASD (17).

Upon the realization of lies in the second stage, due to the relational data on their schema of the environment, the generalization would either occur as a fear of misinformation or factual information. Bagnall et al. (18) discussed two subgroups of children with Autism Spectrum Disorder (ASD): one subgroup was bad at deception yet tended to attempt it, the second subgroup was less likely to attempt to deceive yet were better at doing so. The first subgroup represents a person with a fear of factual information. Due to the avoidance of factual information, they are expected to assimilate many incongruent schemata, leading to a confused perception of their environment and lower neuroplasticity as incongruent ideas will be shown in section 3 to demand more neuroplasticity. This confused perception of their environment in their associative structure leads to unsuccessful attempts at deception. When a child in this subgroup tries to deceive you, I (Long) believe he is trying to share something he enjoys with you. Negative reactions to this deception may lead to a fear of opinions as discussed later in this article.

The second subgroup represents a person with a fear of misinformation. Due to the avoidance of misinformation, they are expected to avoid incongruent schemata, leading to an associative structure that is consistent with their environment and greater neuroplasticity. This consistent associative structure is expected to lead to more successful attempts at deception.

During the third stage, the child becomes aware of opinions. This creates a binary tree splitting with their realization of factual information and misinformation leading to four subgroups. These have been identified by Wheelright et al. (19): Extreme Type S, Extreme Type E, Type B, and the Low-Low Profile.

**Figure 1:**
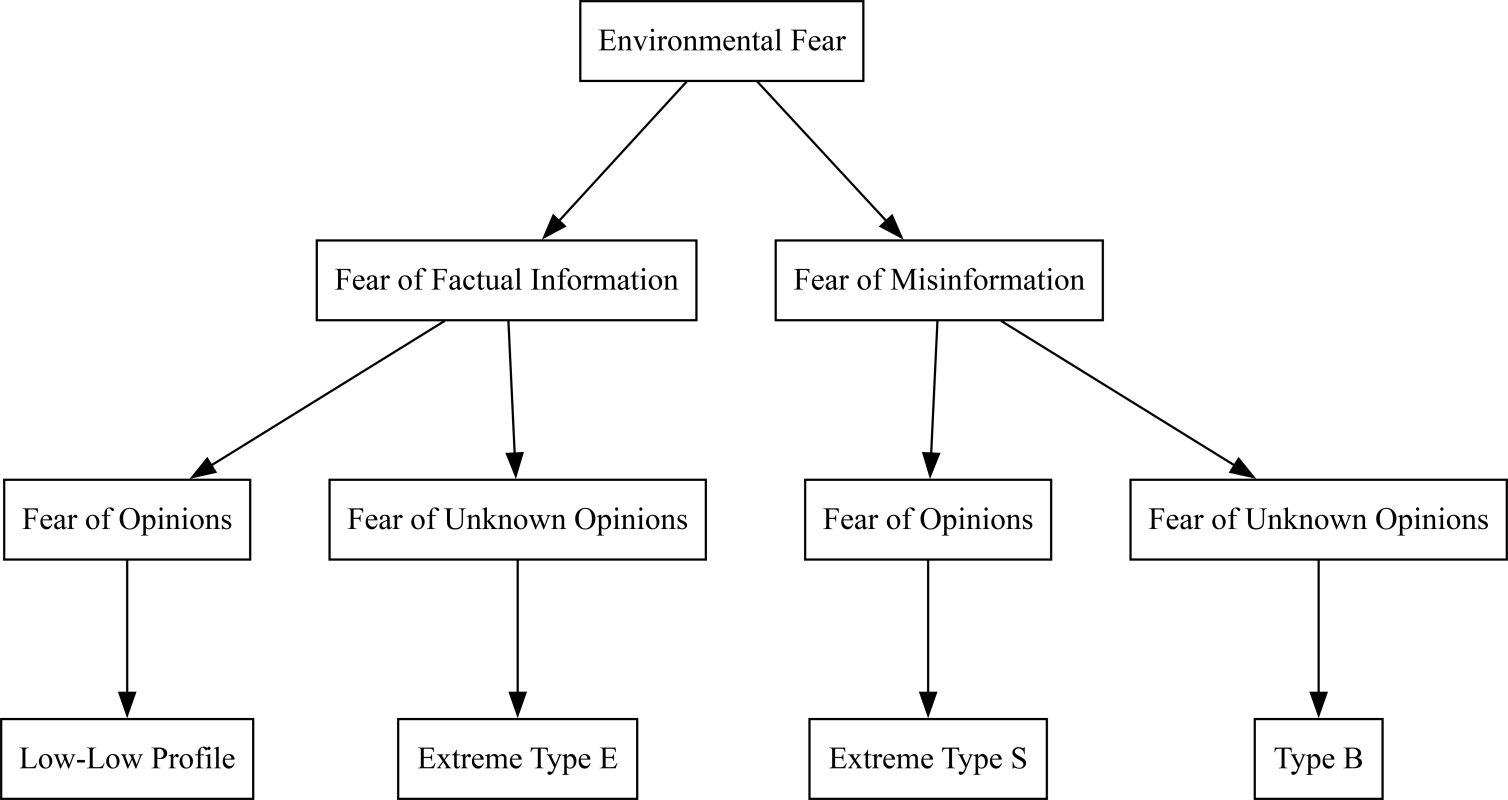
The hypothesized relationship between Goldenfeld’s E/S categorization and the associated fears.

The Extreme Type S is representative of people with a fear of misinformation and a fear of opinions. This subgroup can be expected to have very accurate associative structures regarding their environment. Extreme Type E is representative of people with a fear of unawareness of opinions and factual information. This subgroup can be expected to have very accurate associative structures regarding other people. The Low-Low Profile is representative of people with a fear of factual information and a fear of opinions. While they may seem advisable to address them by hiding your opinions and lying with them, we are concerned that this may lead to the accommodation of either their fear of opinions or their fear of factual information. If they get positive emotions, I (Long) suggest telling the truth to them to avoid schematic restructuring. This is the subgroup I am most concerned about. Since they have so many inconsistent ideas, it is a concern that facing these fears could lead to severe symptoms, or permanent brain damage. Addressing all their assimilated schema in reverse order of acquisition would require an extremely skilled therapist and the work of countless researchers at this time. The final type, Type B, is representative of people with a fear of misinformation and not knowing people’s opinions. This leads to accurate associative structures regarding people and their environment. This relationship is shown in **Figure 1**.

**Figure 2:**
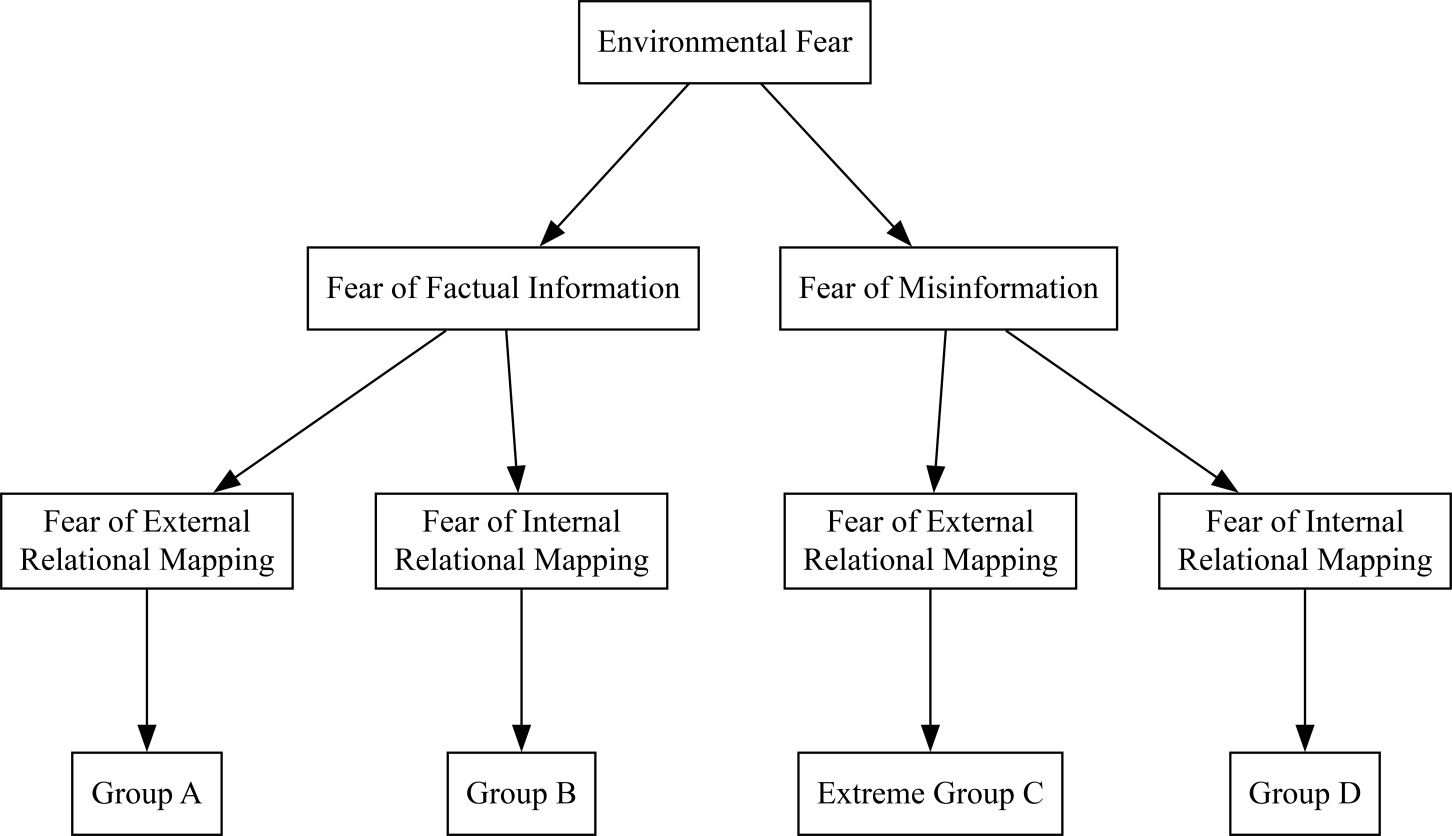
The relationship between the hypothesized subgroups and their associated fears.

At six years of age, there is a change in developmental trajectory of children with ASD (20). This is due to the introduction of flexibility in human cognition (21). What this study showed was the beginning of external relational mapping of schemas. Because this is before the development of the awareness of opinions (or concurrent) we can expect the existence of four subgroups specifically at the age of six. This creates another binary tree with four subgroups: Group A has a fear of factual information and a fear of external relational mapping or a fear of examining their environment. Group B has a fear of factual information and a fear of internal relational mapping or a fear of examining themselves. Group C has a fear of misinformation and a fear of external relational mapping, leading to intense self-examination. And Group D has a fear of misinformation and a fear of internal relational mapping, leading to the intense scrutiny of other people. This relationship is shown in **Figure 2**.

This assumption is borne out by Cognitive Psychology. Upon the realization of external relational mapping, the child becomes aware of external perspectives and at the same time begins to conceptualize his own perspective as something distinct from external perspectives. This leads to generalization to a fear of his perspective or a fear of others’ perspectives. Observation of these subgroups would greatly support this theory.

## 3 Structural outline and effects of schematic restructuring

Upon the arrival of new schemata, the associative structure is strengthened assuming they are not incongruent (22). Incongruent schemata have been shown to create stronger memories (23). This indicates that strong incongruent schemata that are accepted into the associative structure lead to the greater consumption of the same mental resources that lead to neuroplasticity. Due to functional neuroplasticity, we can expect incongruent schemata to consume greater levels of neuroplasticity over time. Due to incongruent schemata leading to a greater reduction in neuroplasticity, we can predict that associative structures seek consistency. With an understanding that the associative structure seeks consistency with your awareness of existence, we can derive a new concept, associative strain which is the force between schema that increases with incongruence and decreases with congruence. This associative strain is expected to lead to a reduction of neuroplasticity over time due to the addition of incongruent schemata to the associative structure. Accommodating a schema that has had multiple schema assimilated to it therefore leads assimilated schema to seek consistency within the new associative structure. As these schemas seek consistency, we can assume they seek to reevaluate their schematic content according to the newly accommodated schema. This is shown in the form of flashbacks to prior memories of when you assimilated schema, as in symptoms of Complex PTSD and Schizophrenia.

Due to schema leading to a reduction in neuroplasticity through utilization, we can assume re-assimilating schema should demand the same if not greater neuroplasticity to assimilate. Due to multiple schema assimilating at once, the brain runs into a deficit of neuroplasticity leading to symptoms of schizophrenia (24, 25). These side effects are temporary (more so if you are dismissing invalid thoughts and delusions). Due to the principles of neuroplasticity (“use it or lose it”) in the brain and the schemas gradually being reassimilated, the brain should recover from schematic restructuring. Due to the associative structure of the brain seeking a consistent worldview, dismissing inaccurate ideas consistently should lead to a quicker recovery rate.

These side effects can be reduced by addressing the schema in reverse order of acquisition. If there are less schema assimilated to a pre-existing schema upon its accommodation, there will be less demand for neuroplasticity. Assuming the new schema is consistent with their awareness, they will benefit from the reduction in utilization of neuroplasticity without the adverse effects (schizophrenia). Due to the gradual “release” of neuroplasticity, we can expect a compounding effect; that is the more you do this, the less risk of aversive side effects. Therapists need to be aware of this.

As for the final hypothesis, due to the avoidant behavior toward factual information shown by some subgroups, we can expect constant assimilation of incongruent schemata to their associative structures. This leads us to believe that these subgroups are at risk for schematic restructuring more so than any other subgroups. Due to the previous hypothesis (that the effects could be reduced by addressing the schemas in reverse order of acquisition), we hypothesize that directly addressing these fears in older children will cause severe schematic restructuring and symptoms of CPTSD and schizophrenia.

## 4 The presence of fear in populations with ASD

### 4.1 Neurobiological support

Autism Spectrum Disorder (ASD) is defined in the Diagnostic and Statistical Manual of Mental Disorders (26) as involving persistent deficits in social communication and interaction across contexts, along with restricted, repetitive patterns of behavior, interests, or activities, with onset in early development and clinically significant impairment (DSM-5). Traditionally, these behaviors are described as developmental differences in cognition and socialization. However, within the framework of the Generalized Environmental Fear Hypothesis for Autism (GEF), they may also be understood as avoidance and coping strategies in response to a unique kind of phobic stimulus: an ongoing awareness of one’s own environment. This reframing does not dismiss existing neurodevelopmental accounts but instead highlights how chronic dysregulation of fear systems might contribute to what we already observe. Neuroimaging findings in ASD subjects show alterations in regions commonly linked to fear and salience processing, including reduced resting state connectivity between the amygdala and anterior cingulate cortex (27) and atypical activation of the insula during attentional tasks (28). Large-scale rs-fMRI studies also confirm widespread atypical connectivity in the salience network, including the anterior insula and ACC, reinforcing the view that ASD involves chronic dysregulation of self-related salience processing (29). Such findings are consistent with sustained engagement of fear related circuitry, as might be expected if the perceived threat were internal and continuous.

Specific phobia, in contrast, is defined in the DSM-5 as a marked and disproportionate fear of a specific object or situation that reliably provokes immediate anxiety, results in avoidance, and produces significant distress or impairment over time (26). In these conditions, the fear circuit is activated episodically in the presence of discrete stimuli. Under GEF, the difference is that the feared stimulus is not external but instead tied to self-awareness, making avoidance impossible and dysregulation chronic. Research on specific phobia consistently demonstrates hyperactivation of the amygdala, anterior insula, and anterior cingulate cortex during stimulus exposure, alongside diminished prefrontal regulatory function (30). Recent structural MRI work has also shown gray and white matter alterations across cortico-subcortical networks in phobia, particularly in prefrontal regions overlapping with ASD findings, suggesting convergent patterns of dysregulation in fear regulatory circuitry (31). These same regions have been implicated in ASD, suggesting a potential neurobiological overlap worth considering.

Shared neurotransmitter involvement adds further nuance to this comparison. For instance, perinatal exposure to selective serotonin reuptake inhibitors (SSRIs) has been shown to alter amygdala and anterior cingulate circuitry and heighten innate fear reactivity in both human and animal models (32). Similarly, hypoxia ischemia during development disrupts GABAergic interneuron maturation in the amygdala (gamma-aminobutyric acid, the brain’s primary inhibitory neurotransmitter), impairing inhibitory control and leaving fear responses less regulated (33). Reviews of inhibitory system development further emphasize that disruptions in GABAergic signaling are critical to the maturation of fear regulation circuits, a mechanism plausibly shared between phobia and ASD (34). While these findings do not prove equivalence between ASD and specific phobia, they illustrate how early disruptions in serotonin and GABA systems could plausibly sustain heightened fear responses to internal or abstract stimuli.

At the circuit level, both ASD and phobia consistently involve the amygdala, insula, and anterior cingulate network. In phobia, activation of this network occurs when a specific external threat is encountered. In ASD, GEF suggests the same circuitry may be persistently engaged because the perceived source of fear, the environment, cannot be escaped. Deficits in prefrontal regulation, such as reduced medial orbitofrontal cortex volume in panic disorder with agoraphobia (35) and reduced prefrontal activation during socio-emotional processing in ASD (28), point to a shared difficulty in regulating fear responses once they are triggered. Early developmental evidence further shows that infants at elevated risk for ASD, such as those born preterm, display atypical lateralization of fronto-limbic networks including the amygdala and orbitofrontal cortex, indicating that vulnerabilities in these circuits emerge very early (20).

Perinatal trauma may not only sensitize the fear circuit but also influence its developmental trajectory. During the first six years of life, when the ASD brain demonstrates rapid neural growth and synaptic overproduction, heightened activation within amygdala and cingulate pathways may transiently enhance sensory vigilance and responsiveness to environmental cues, an adaptation consistent with the accelerated neural maturation observed in early ASD development (20). As cortical inhibitory systems dependent on GABAergic maturation begin to emerge, however, this same hyperactivation may become maladaptive, maintaining an elevated baseline of excitability within fear regulatory networks (33, 34). Early disturbances in serotonin signaling, hypoxia, or inflammatory processes could therefore consolidate a state of persistent salience dysregulation, transforming initially adaptive hyperreactivity during early childhood into a long-term impediment to regulatory balance (36). In this way, perinatal disruptions provide a plausible bridge between transient developmental overactivation and the enduring patterns of fear circuit engagement that characterize ASD.

Cellular and genetic evidence also point to partial convergence. Environmental risk factors for ASD, including maternal inflammation and metabolic conditions, have been linked to heightened amygdala excitability and oxidative stress (33), both of which can predispose to persistent fear responses. More recent reviews confirm that maternal immune activation and oxidative stress remain central to ASD pathophysiology, reinforcing the plausibility of persistent hyper-reactivity within fear circuits (36). While genome wide studies on specific phobia are limited, broader anxiety spectrum findings suggest overlapping pathways related to excitatory inhibitory balance and synaptic plasticity. Twin studies further indicate that genetic factors account for a substantial proportion of variance in autism likelihood (heritability estimates ranging from roughly 64 to 91 percent) based on markedly higher concordance among monozygotic than dizygotic twins (37). However, it is important to note that monozygotic twins have been found to share not only their genome but also many perinatal physiological experiences, including shared placental environments and exposure to hypoxia, inflammation, and delivery-related stress (38, 39). From the perspective of GEF, these events could act as conditioning stimuli that sensitize the fear circuit. This shared exposure may partly account for the high concordance rates in identical twins, suggesting that what appears as genetic heritability may reflect an interaction between genetic susceptibility and common perinatal trauma. These parallels suggest that common vulnerabilities in fear regulation may underlie both conditions, though ASD may represent a unique and more generalized manifestation.

Neuroimaging studies further reinforce these parallels. Grey matter reductions in prefrontal regulatory regions are observed in both phobia and ASD, consistent with impaired top down modulation of fear (28, 30). Functionally, individuals with phobia demonstrate amygdala hyperactivation to specific feared stimuli, while individuals with ASD show impaired discrimination between safe and threatening cues, a finding that may reflect generalization of fear to internal or self-related stimuli (40). Large-scale fMRI meta-analyses of fear conditioning also demonstrate robust engagement of the anterior cingulate, insula, and dorsolateral prefrontal cortex, with reduced ventromedial prefrontal activation during safety learning, directly paralleling findings in both phobia and ASD (41). Safety learning and extinction also appear disrupted in both conditions: prolonged extinction in phobia (42) parallels reduced safety learning in ASD (40, 43). Developmental work further shows that extinction retention is fragile, with adolescents in particular displaying stronger amygdala fear responses during extinction tests, a pattern consistent with the persistence of conditioned fear seen in ASD (44). Both sets of findings suggest entrenched conditioning of fear responses, though triggered by different types of stimuli.

Oscillatory dynamics offer additional clues. Fear states are known to modulate theta gamma coupling between the amygdala and medial prefrontal cortex, while abnormal beta and gamma patterns correlate with anxiety severity and phobia classification. Comparable abnormalities in network oscillations have been described in ASD, pointing to shared dysfunction in salience signaling and fear regulation. Adults with autism also demonstrate exaggerated social fear learning, showing stronger autonomic responses to observed threats compared to controls, evidence that fear generalization processes are intensified rather than diminished in ASD (45). Together, these findings suggest that persistent salience dysregulation may operate across both conditions, but in ASD the trigger may be self-related awareness rather than discrete external stimuli.

Intervention studies offer a practical perspective on these similarities. SSRIs, commonly prescribed in anxiety disorders, target serotonergic pathways also implicated in ASD risk and fear circuitry development (32). Exposure based cognitive behavioral therapy (CBT), the gold standard for phobia, is designed to strengthen safety learning and weaken maladaptive fear associations. If GEF holds true, then carefully adapted exposure methods targeting self-related or existential cues may offer benefit to individuals with ASD by helping to recalibrate persistent fear responses.

Despite these parallels, caution is warranted. The amygdala, insula, and anterior cingulate network is transdiagnostic and implicated in many psychiatric conditions, meaning overlap alone cannot establish identity. Developmental risk factors such as inflammation, SSRI exposure, or excitatory inhibitory imbalance may alter fear circuitry without necessarily producing a phobia of environment. Moreover, ASD is highly heterogeneous, not all individuals show anxiety-like processing, and many function without significant fear related difficulties. For these reasons, GEF should be considered a hypothesis that highlights one possible explanatory mechanism rather than a replacement for existing models.

### 4.2 Falsifiability

We believe the primary method for falsification of GEF involves identification of the four subgroups listed in section 2. Another method involves testing a population for the presence of a fear using EMDR and memory formation measures (46, 47).

Fear is measured primarily through subjective measures such as subjective units of distress, with objective physiological indicators complementing the subjective measures. EMDR is used to reduce fear in the participant with the goal of remembering the experience less vividly. As a result, it desensitizes the impact of the fearful experience decreasing the autonomic fear response when recalling. Similarly, individuals can be expected to show a weakened fear response while memorizing through different measures. Individuals with ASD will have greater variability in results while using EMDR due to differences in memory, attention, sensory input, and emotional processing.

Assuming EMDR can serve to suppress the level of fear in a subject, we can then use memory formation measures to determine the level of fear in an individual. Therefore, the theory of GEF could be supported or disproven based on the results of using the therapeutic method to test and measure memory formation.

Another method of falsification is to measure neuroplasticity between the subgroups we expect to fear factual information and the subgroup expected to fear misinformation. Due to the assimilation of incongruent schema, we expect a fear of factual information to show lessened neuroplasticity.

The final method of falsifiability is lying to subgroups suspected of having a fear of factual information. Another extension of this idea is to teach children from this subgroup of solipsism, the impact of this is difficult to know. We expect that misinformation should be more acceptable to people from the subgroups believed to fear factual information.

## 5 Limitations

This article is susceptible to confirmation bias due to its exploratory nature.

## 6 Discussion

While at this time, nothing has been confirmed regarding this hypothesis, this article has shown support for each of its hypotheses as well as methods for falsification. While this article does not draw connections between many of the current models of Autism Spectrum Disorder (ASD), a future article may make these observations. For the field of Autism research, this article should lend itself to new avenues of research, particularly observing fear generalization as a potential correlated factor. Should this article prove unfalsifiable, this will provide support for neurobiological and cognitive perspectives described. Due to the exploratory nature of this article, it is susceptible to confirmation bias. While this article has provided support for the benefits of Cognitive Behavioral Therapy (CBT) as well as warnings of the suspected risks of CBT, more research is necessary to confirm or refute these potential risks. While this may cause some people to avoid CBT, it is our belief that ultimately CBT will continue to rise in popularity due to its potential benefits.

## 7 Conclusion

GEF provides a framework for better understanding ASD. We believe this hypothesis to be well supported, though not conclusively. First we described the mechanism by which the brain is neurologically altered in populations of ASD, then we showed similarities between the developmental trajectories of neurotypical populations and populations with ASD. Finally we neurologically supported the claim that populations with ASD were in a constant state of fear. I expect this paper to have broad implications, not just for the field of Autism Research, but for neurodevelopmental research, schema theory research, fear and salience network research, as well as identifying other disorders associated with fear.

## Author’s note

Published under a pseudonym at the author’s request for safety reasons. All declarations and affiliations have been verified and are held in confidence by the journal.

## Data Availability

The original contributions presented in the study are included in the article/supplementary material. Further inquiries can be directed to the corresponding author.
